# Supplementary material for: Association of comorbidity between Opisthorchis viverrini infection and diabetes mellitus in the development of cholangiocarcinoma among a high-risk population, northeastern Thailand
Source: PLoS Negl Trop Dis. 2021 Sep 20;15(9):e0009741. doi: 10.1371/journal.pntd.0009741 (PMC8452023; doi:10.1371/journal.pntd.0009741)
Supplement: Attachment — Submitted filename: Response to reviewers_20210620.docx [file pntd.0009741.s001.docx]

Dear Editor,

Thank you very much for giving us the opportunity to improve and resubmit our manuscript "Association of comorbidity between *Opisthorchis viverrini* infection and diabetes mellitus in the development of cholangiocarcinoma among high risk population, northeastern Thailand"

Please find enclosed the revised manuscript for further consideration. The manuscript has been revised according to the comments raised by the reviewer to the best of our ability.

Changes to the manuscript are highlighted in yellow. Please find a detailed reply to the reviewer comments attached with this revision. We would like to thank the reviewer for the constructive and competent criticism, and we hope that our manuscript will be acceptable for publication in PLOS Neglected Tropical Diseases.

**Response to the reviewers**

**Reviewer: 1**

Comments to the Author

**Methods**

**Reviewer #1:** Feasible

**Reply:** Thank you for your comment.

**Results**

**Reviewer #1:** Clear presentation

**Reply:** Thank you for your comment.

**Conclusions**

**Reviewer #1:** Somewhat weak

**Reply:** Thank you for your comment. We have now strengthened our Discussion and Conclusions

**Summary and General Comments**

**Reviewer #1:** General Comments

This is a large scale cross-sectional retrospective study on the effects of co-morbidity of Ov infection and DM on cholangiocarcinogenesis.

The story is smiple and the experimental design is straightforward. The results are clear-cut.

In contrast, the MS is lengthy, wordy with many repetitions. Introduction and Discussion can be shortened considerably to make MS much more impressive.

**Reply:** Thank you for your comments and suggestions. We have extensively revised the introduction and discussion sections as you suggested.

**Reviewer #1:** Specific Comments

Although this MS has a co-author of native-English using scientist, there are numerous typos and English usage problems, although those does not affect the scientific quality of this work.

**Reply**: A native English speaker has reviewed the entire manuscript and corrected usage problems as well as the more specific remarks below.

1) Typically, for study areas, “Northeast of Thailand” in the title, but “Northeastern Thailand”, “Northeast Thailand”, “Thailand’s Northeast region” in other places. I believe the Thai government’s official expression is “the Northeast Thailand” and “northeastern Thailand”. Consistency is required.

**Reply:** Thank you for your suggestions. To make it consistent, we have changed the word to “northeastern Thailand” in our manuscript.

2) Opishorchis viverrini is the name of parasite, whereas Diabetes mellitus is the name of disease. Whenever you are talking about co-morbidity, or combination of O. viverrini infection and DM, you should use “opisthorchiasis” or “O. viverrini infection” instead of “O. viverrini”

**Reply:** Thank you for pointing this out. To make it clear, we have replaced the word “*O. viverrini* infection” in all sections in our manuscript.

Discussion section is too much repeat of the results. What is important is how DM and Ov infection mutually affect to lead carcinogenesis. Please modify discussion to provide more about possible mechanisms of co-morbidity of opisthorchiasis and DM on CCA genesis.

**Reply:** Thank you for your suggestions. We have modified the discussion as you suggested in the page 16, line numbers 346.

**Reviewer: 2**

Comments to the Author

**Methods**

**Reviewer #2:** = objective clearly stated

= study design is appropriate

**Reply:** Thank you for your comments.

= Hypothesis missing. proposed to clearly spell-out the hypothesis in the last paragraph of the instroduction

**Reply:** Thank you for your suggestion. We have added the text in to the Introduction section line 141 giving more details of hypothesis as follows:

***Page 6, line number 141:*** We hypothesize that the group who were infected with *O. viverrini* and who also had a positive DM diagnosis will have a higher risk of CCA than other groups.

= sample size is fixed as CASCAP data was used

**Reply:** Thank you for your comment.

= statistical analysis should be reviewed by statistician

**Reply:** Thank you for your suggestion, the statistical analysis for our study have already been reviewed by statistician.

= yes, data originates from large-scale ongoing screening program. Ethics statement is not given in manuscript. Should be added.

**Reply:** Thank you for your suggestion. We have added the text in to the Materials and methods section under the heading ‘Ethics statement’ line 148 giving more details of Ethics statement as follows:

***Page 7, line number 148:*** The data were requested from Cholangiocarcinoma Screening and Care Program (CASCAP). The CASCAP data collection was conducted according to the principles of Good Clinical Practice, the Declaration of Helsinki, and national laws and regulations about clinical studies. It was approved by the Khon Kaen University Ethics Committee for Human Research under the reference number HE551404.

please address the following important points:

= please provide for all variables used in this analysis a clear definition:

1. outcome: please state whether CCA was defined as histologically positive CCA or also other assessments.

**Reply:** Thank you for your suggestion. We have added the text in to the Materials and methods section under the heading ‘Study outcome and independent variables’ line 190 giving more details of study outcome as follows:

***Page 8, line number 190:*** The primary outcome for this study was CCA which was defined as histologically positive and categorized into two groups (no/yes).

2. O. viverrini infection: state clearly that this is a reported history of O. viverrini infection. Was the question restricted to a time period or any prior diagnosis.

**Reply:** Thank you for your question. The question in the CASCAP questionnaire was not restricted to a time period or any prior diagnosis. The question is "Have you ever been found to be infected by liver fluke?".

3. Diabetes assessment was also reported by the patient. What exactly was asked. Ever diagnosed? Has the current treatment been included

**Reply:** Thank you for your questions. The question for diabetes assessment is history of diagnosis. The question is "What diseases have you been diagnosed with?", and with this question, if the participants check the choice of "Diabetes" means that they have been diagnosed.

4. smoking: please clarify if current smoking, ever smoking, smoking quantity etc.

**Reply:** Thank you for you suggestion. The smoking variable is actually history of smoking (no/yes, current or previous).

5. drinking alcohol: please clarify what is considered drinking alcohol? is linked with quantity etc. or provide question.

**Reply:** Thank you for you suggestion. The drinking alcohol variable is defined as history of drinking (no/yes, current or previous).

6. eating raw fish: please specify what exactly was asked to participant (ever eaten raw fish, fish sauce, fermented fish etc.)

**Reply:** Thank you for you suggestion. The eating raw fish variable is defined as history of eating (no/yes, current or previous).

For the reviewer comments number 2-6, to make it clear, we have added the text in to the Materials and methods section under the heading ‘Study outcome and independent variables’ line 206 giving more details of independent variables as follows:

***Page 9, line number 206:*** history of smoking cigarettes defined as no/yes (current or previous), drinking alcohol defined as no/yes (current or previous), history of eating raw fish defined as no/yes (current or previous), and history of praziquantel treatments (PZQ) defined as never/one time/two times/three times and more.

= please improve the statement of the statistical analysis in order to be:

When performing the adjusted analysis, did the authors adjust always for all the other variable. In Table 2 is it correct that for each aOR provided the 9 other variables were used for adjustment. Or, did the author conduct one multivariable analysis and report the adjusted results?

**Reply:** Thank you for your questions. We adjusted always for all other variables for the adjusted analysis. In Table 2, each aOR was obtained from a multivariable analysis which was adjusted for all variables in the table.

**Results**

**Reviewer #2:** = results presented match the analysis plan
= result presentation can be improved:
**1. In descriptive summary**
- please add also overall prevalence of Opisthorchis infection, DM and CCA.

**Reply:** Thank you for your suggestion. We have added the text in to the Results section under the heading ‘Descriptive summary’ line 237 giving more details of *O. viverrini* infection and DM as follows:

***Page 10, line number 237:*** Among study participants, overall prevalence of *O. viverrini* infection was 32.4%, and overall prevalence of DM was 8.2%.

For the prevalence of CCA, we already reported in Table 2 and the subheading ‘Rate and association summary’ line 260 saying that “From a total of 263,776 participants, the overall rate of CCA was 0.36%”.

**2. Table 1**
- please add in all columns a column-percent in brackets as it is done in the "Total" column. This will add more numbers to the table but provides the reader an easier insight into the data which currently difficult because the reported number of observations in the subgroups are huge.

**Reply:** Thank you for your comment. We have added the column-percent in Table 1.

**3. Table 2**
This is the key results table of the manuscript. Hence it is worthy to add elements to provide easier insights in the data (landscape presentation might be necessary). Suggested to:
- add column percent in the "number" column

**Reply:** Thank you for your suggestion. We think that the column percent is not necessary in this table, as we already reported the column percent in Table 1.

- insert a column next to "number" column (left of "%CCA" column) in which absolute number of CCA cases are reported

**Reply:** Thank you for your suggestion. We have added the column CCA cases in Table 2.

- insert two column in which 95% Ci and p-value of cOR are reported

**Reply:** Thank you for your suggestion. We have added the two columns, 95% CI and p-value of cOR in Table 2.

- insert a column in which 95% Ci and p-value of aOR are reported

**Reply:** Thank you for your suggestion. We already reported 95% CI and p-value of aOR in Table 2.

- please clarify the role of the reported p-value

**Reply:** The p-value reported in Table 2 and 3 indicates the results of a chi-squared test measuring differences in proportions. For example in the first row of table 2 the p-value indicates that there is a statistically significant difference in the proportions having CCA between the OV yes and the OV no groups. If the p-value is less than 0.05, the factor is statistically significantly related to CCA.

**4. Table 3**
- please modify the table in analogy of table 2

**Reply:** Thank you for you suggestion. Actually Table 3 is the key results and we have re-designed and added all information needed including crude OR, adjusted OR, 95% CI, and p-value for each categories of combination of *O. viverrini* infection and DM.

- the content of this table is largely represented by Fig 6. Therefore, table 3 could be moved to a supplementary file

**Reply:** Thank you for your suggestion. Figure 6 is mainly included to compare the magnitude of association reported in table 2 and table 3 between the factors of OV infection alone, DM alone, and combination of OV infection and DM. We decided to keep table 3 in the main text as this table presents the main results of the study.

**5. Fig 1**
- can the exact dates of used registration period be given (1 Jan 2013 - 31 Dec 2019)?

**Reply:** Thank you for your suggestion. We have added the exact dates of registration period as 9 Feb 2013 - 31 Dec 2019 in to Fig 1.

**6. Fig 2**
- please use also colors.

Reply: Thank you for your suggestion. We have changed Fig 2 as colors.

- please add overall section with prevalence of O. viverrini infection and DM

Reply: Thank you for your suggestion. We have added section of overall prevalence of *O. viverrini* and DM in to Fig 2.

**7. Fig 3**
- please use also colors

**Reply:** Thank you for your suggestion. We have changed Fig 3 as colors.

**8. Fig 4**
- please use also colors

**Reply:** Thank you for your suggestion. We have changed Fig 4 as colors.

- suggested to add overall section with CCA incidence in Ov alone and DM alone

**Reply:** Thank you for your suggestion. We have added section of overall CCA incidence in *O. viverrini* and DM in to Fig 4.

**9. Fig 5**
- please use also colors

**Reply:** Thank you for your suggestion. We have changed Fig 5 as colors.

- suggestion: it might be clearer with the smoothed age-prevalence curves

**Reply:** Thank you for your suggestion. We have made the curves smoothed for the Fig 5.

**10. Fig 6**
- very nice! It could replace Table 3, see above

**Reply:** Thank you for your comment. We have responded regarding replacing Table 3 above.

- please provide exact aOR above the rhomboid symbol

**Reply:** Thank you for your suggestion. We have added exact aOR above the rhomboid symbol for categories in Fig 6.

11. All tables and figures
- please provide in addition to the title a descriptive text with details in order to make the table/figure self-explanatory

**Reply:** Thank you for your suggestion. We have added the text to make the table/figure self-explanatory for all tables and figures.

**Conclusions**

**Reviewer #2:** The authors report on a CCA risk increase when Ov infection and DM is present and was highest when both exposure were present. However, the discussion regarding the internal validity of the study is rather weak. The recall bias is mentioned in the limitations but could be addressed more clearly.

**Reply**: We have added some more clarification in the limitations section concerning recall bias.

Further other bias as selection bias is likely to present. In Fig 1 clearly shows that almost 1 million people were excluded from the analysis. Could they have resulted in a selection bias.

**Reply:** The reviewer is correct that a large number of potential participants were excluded. These were people who had missing data for one of the variables of interest. We feel however that in a study of this size the potential bias introduced here will be minimal. There is no systematic way in which those excluded were different from those included except for those missing variables.

In addition, study participants consisted of "screening" and "walk-in" group patient groups. In how far could this fact have influenced the observed associations?

**Reply:** The screening and walk-in groups are different in that the walk-in group purposively joined the study because of perceived risk. This means that the likely prevalence of CCA in this group will be higher than for the screening group. However, assigning proportions of people to these two groups was not a part of the study design, we did not set out to recruit a particular number of people from each group. These are simply the people who have joined the study and the results reflect the number who self-reported symptoms and registered for CASCAP. This cannot really be considered bias as the study is not proposing external validity.

Finally, existing evidence on the underlying biological mechanisms is given in the introduction. However, it would be great if this section in the introduction (lines 118-125) could be moved to the discussion and could be elaborated on in more detail. It is interesting that one cited article (Tsai et al, Int J Cancer, 2015; ref 14) reports on a negative association between DM and CCA.
Consequently, the conclusion of the article could be adapted to this discussion part and tuned down a bit. However, the public health message is nicely stated and more attention should be given to the multi-morbidity groups.

**Reply:** Thank you for your comments and suggestions. We have revised according to your suggestion.

**Editorial and Data Presentation Modifications**

**Reviewer #2:** = Please, carefully English edit the text. E.g. "had O. viverrini positive" or "had DM positive" is not correct English; line 101: drop "old", line 133-4: suggest to revise sentence; line 173: pathological or histological? line 163-4: Ov infection prevalences are higher in Laos than in NE Thailand; reported CCA incidence are worldwide highest in NE Thailand;

**Reply:** Thank you for your comments and suggestions. We have revised according your suggestion.

= Please check references and edit according to the journals recommendations. Italicize genus/species names.

**Reply:** Thank you for your suggestion. We have edit the references according to the journal recommendations.

= please check sentence on line 105: meaning seems inadequate. Most Ov re-infection are due to raw/fermented fish consumption

**Reply:** Thank you for your suggestion. We have edited according to your suggestion.

**Summary and General Comments**

**Reviewer #2:** Congratulations! This is a extremely nice and most valuable report. It is based on a unique data base, CASCAP. The conclusions on the study are of importance for Southeast Asia, particularly because of O. viverrini rates are high in many places and diabetes prevalence is in the increase.

**Reply:** Thank you for your reviews and comments.

Best regards,

Apiporn T Suwannatrai and co-authors
