# Supplementary material for: Association of comorbidity between Opisthorchis viverrini infection and diabetes mellitus in the development of cholangiocarcinoma among a high-risk population, northeastern Thailand
Source: PLoS Negl Trop Dis. 2021 Sep 20;15(9):e0009741. doi: 10.1371/journal.pntd.0009741 (PMC8452023; doi:10.1371/journal.pntd.0009741)
Supplement: Attachment — Submitted filename: Response to Editor and reviewers.docx [file pntd.0009741.s002.docx]

**Response to Editor and Reviewers**

Dear Editor,

We thank you very much for the comments and suggestions. The comments and suggestions are valuable and very helpful for revising and improving our manuscript. We have made revisions according to reviewer’s comments and suggestions, as described in the authors’ response. Please find enclosed the revised manuscript for further consideration. The manuscript has been revised according to the comments raised by the reviewer to the best of our ability. Changes to the manuscript are tracked changes denoting where the text has been changed. Please find a detailed reply to the reviewer comments attached with this revision. We would like to thank the reviewer for the constructive and competent criticism, and we hope that our manuscript will be acceptable for publication in PLOS Neglected Tropical Diseases.

Yours sincerely,

Apiporn T. Suwannatrai and co-authors

**Response to reviewers**

**Manuscript Number:** PNTD-D-21-00483R1

**Title:** Association of comorbidity between *Opisthorchis viverrini* infection and diabetes mellitus in the development of cholangiocarcinoma among a high risk population, northeastern Thailand

**Methods**

Reviewer #1: (No Response)

Reviewer #2: please see general comments

**Response:** Thank you for your comments.

**Results**

Reviewer #1: (No Response)

Reviewer #2: Authors have improved the presentation of the results.

**Response:** Thank you very much.

In the result tables of the risk analysis p-values are provided. Please specify from which statistics the p-values are (from the LR chi2 or z-statistics?). Please specify in the table legend. If the p-value is from z-statistics then it should be given on the same row as the OR and the 95% CI of the OR.

**Response:** Thank you for your suggestions. We added the sentence as “from likelihood- ratio chi-square tests” in the legend of Table 2 in the page 12, line numbers 261-262, and Table 3 in the page 14, line numbers 296-297.

The Figures and Tables have been improved and are now self-explanatory. However, the explanatory text need to be English edited.

**Response:** Thank you very much. We have extensively revised the English in the text by asking Prof. David Blair, an English scientific expert, to edit English.

**Conclusions**

Reviewer #1: (No Response)

**Reviewer #2: good and have been improved**

**Response:** Thank you very much.

--------------------

Editorial and Data Presentation Modifications?

Use this section for editorial suggestions as well as relatively minor modifications of existing data that would enhance clarity. If the only modifications needed are minor and/or editorial, you may wish to recommend “Minor Revision” or “Accept”.

Reviewer #1: (No Response)

Reviewer #2: It is still necessary that the text is English edited.

**Response:** Thank you very much. We have extensively revised the English in the text by asking Prof. David Blair, an English scientific expert, to edit English.

Please, check Table 3. The presentation is confusing. Explanations of the adjustments should be given in the legend. The p-value seems to be indicated in the wrong row. The crude and adjusted OR should be always corrected abbreviated (aOR, cOR) and the abbreviations consistently used (in this table and in the entire manuscript). Please also introduce the aOR and cOR in the analysis section of the methods.

**Response:** Thank you for your suggestion. We have checked and created new Table 3, corrected abbreviated (aOR, cOR), and the abbreviations consistently used in the table and in the entire manuscript. We introduced the aOR and cOR in the analysis section of the methods.

--------------------

**Summary and General Comments**

Use this section to provide overall comments, discuss strengths/weaknesses of the study, novelty, significance, general execution and scholarship. You may also include additional comments for the author, including concerns about dual publication, research ethics, or publication ethics. If requesting major revision, please articulate the new experiments that are needed.

**Reviewer comments:**

**Reviewer #1: Comments to PNTD-D-21-00483R1**

This revised version is acceptable after some corrections for English usages. Especially attention is required for the usage of the abbreviations like OV and DM for the group names. Consistent use of abbreviation is required.

**Response:** Thank you for your comments and suggestions.

I. 89: were >> are

**Response:** Thank you for your suggestion. We changed from “were” to “are” in the page 4, line number 88.

I.94: people >> those

**Response:** Thank you for your suggestion. We have rewritten this sentence from “The study findings revealed that people who were infected with *O. viverrini* and had diabetes mellitus (DM) were highly associated with CCA with a higher risk than **people** who were only infected with *O. viverrini* or had DM alone.” to “We found that infection with *O. viverrini* and a diagnosis of diabetes mellitus (DM) was highly associated with CCA. The risk of CCA in individuals with both of these conditions was higher than in individuals with only one of them.” in the page 4, line number 92-94.

I.103: delete “and” after Thailand

**Response:** Thank you for your suggestion. We deleted “and” after Thailand in the page 5, line number 101.

I.107: delete “in Thailand”

**Response:** Thank you for your suggestion. We deleted “in Thailand” in the page 5, line number 105.

I.110: by >> with

**Response:** Thank you for your suggestion. We changed from “by” to “with” in the page 5, line number 108.

I.120: in >> of

**Response:** Thank you for your suggestion. We changed from “in” to “of” in the page 5, line number 117.

I.119-120: change to “has shown that, although statistically not significant, DM was associated with shorter survival of CCA patients [17].

**Response:** Thank you for your suggestion. We changed to “has shown that, although statistically not significant, DM was associated with shorter survival of CCA patients [17]” in the page 5, line number 117.

I.121: reported >> revealed

**Response:** Thank you for your suggestion. We removed the sentences as “A recent survival study in China **reported** that patients with preoperative hyperglycemia had a shorter survival time after gallbladder surgery [18]. These findings are biologically plausible. Hyperglycemia can stimulate the proliferation of cancer cells, negatively impacting survival for CCA patients.” in the page 5, line number 117.

I.123: delete “the”

**Response:** Thank you for your suggestion. We deleted “the” and removed the sentences as “A recent survival study in China reported that patients with preoperative hyperglycemia had a shorter survival time after gallbladder surgery [18]. These findings are biologically plausible. Hyperglycemia can stimulate **the** proliferation of cancer cells, negatively impacting survival for CCA patients.” in the page 5, line number 117.

I.135: underway as the

**Response:** Thank you for your suggestion. We changed from “underway” to “underway run by” in the page 5, line number 127.

I.148: requested from >> provided from

**Response:** Thank you for your suggestion. We changed from “requested from” to “provided from” in the page 6, line numbers 139.

I.193: This group were >> Those included in this group were

**Response:** Thank you for your suggestion. We changed from “This group were” to “Those included in this group were” in the page 8, in the page 180

I.196: negative results >> negative results group

**Response:** Thank you for your suggestion. We changed from “negative results” to “negative for” in the page 8, line number 182.

I.199: data on >> data of

**Response:** Thank you for your suggestion. We changed from “data on” to “data of” in the page 8, line number 186.

I.202: diagnosis with DM >> diagnosis of having DM

**Response:** Thank you for your suggestion. We changed from “diagnosis with DM” to “diagnosis of DM” in the page 8, line number 189.

I.202: in to >> into

**Response:** Thank you for your suggestion. We changed from “in to” to “into” in the page 8, line number 189.

In the l.201-205 in the M&M section, the authors have defined 4 groups of participants using abbreviation of OV for O. viverrini infection and DM for diabetes mellitus.

In the Results section, the group names have been used inconsistently. Typical example can be seen in l.237-245, but inconsistency throughout in the Results and Discussion.

Consistent use of abbreviation is required.

**Response:** Thank you for your suggestion. We have changed all abbreviations as consistent in the results and discussion parts.

Reviewer #2: This is a very interesting study. The authors have addressed all the points raised in my first assessment.

**Response:** Thank you very much.
